# Metabolism-Disrupting Chemicals and the Constitutive Androstane Receptor CAR

**DOI:** 10.3390/cells9102306

**Published:** 2020-10-15

**Authors:** Jenni Küblbeck, Jonna Niskanen, Paavo Honkakoski

**Affiliations:** 1 A.I. Virtanen Institute for Molecular Sciences, University of Eastern Finland, P.O. Box 1627, FI-70210 Kuopio, Finland; jenni.kublbeck@uef.fi; 2 School of Pharmacy, University of Eastern Finland, P.O. Box 1627, FI-70210 Kuopio, Finland; jonna.niskanen@uef.fi; 3 Division of Pharmacotherapy and Experimental Therapeutics, Eshelman School of Pharmacy, University of North Carolina at Chapel Hill, Campus Box 7569, Chapel Hill, NC 27599-7569, USA

**Keywords:** endocrine disruption, metabolic disruptors, constitutive androstane receptor, NR1I3, glucose metabolism, lipid metabolism

## Abstract

During the last two decades, the constitutive androstane receptor (CAR; NR1I3) has emerged as a master activator of drug- and xenobiotic-metabolizing enzymes and transporters that govern the clearance of both exogenous and endogenous small molecules. Recent studies indicate that CAR participates, together with other nuclear receptors (NRs) and transcription factors, in regulation of hepatic glucose and lipid metabolism, hepatocyte communication, proliferation and toxicity, and liver tumor development in rodents. Endocrine-disrupting chemicals (EDCs) constitute a wide range of persistent organic compounds that have been associated with aberrations of hormone-dependent physiological processes. Their adverse health effects include metabolic alterations such as diabetes, obesity, and fatty liver disease in animal models and humans exposed to EDCs. As numerous xenobiotics can activate CAR, its role in EDC-elicited adverse metabolic effects has gained much interest. Here, we review the key features and mechanisms of CAR as a xenobiotic-sensing receptor, species differences and selectivity of CAR ligands, contribution of CAR to regulation hepatic metabolism, and evidence for CAR-dependent EDC action therein.

## 1. Introduction

Endocrine-disrupting chemicals (EDCs) are natural or synthetic compounds that alter functions of the endocrine system and as a consequence, cause adverse health effects in an intact organism or its progeny or subpopulations, e.g., via interactions with nuclear receptors (NRs) and other targets of endogenous hormones and other signaling molecules [1,2]. The synthetic EDCs are structurally diverse, often lipophilic substances capable of bioaccumulation (persistent organic pollutants). Examples include plasticizers, pesticides, fungicides, and various polyhalogenated organic compounds present in consumer products, environment, or exposures from industrial sources [1]. EDCs may interfere with regulation of body homeostasis by mimicking, suppressing, or altering the normal physiological responses. These adverse actions can be mediated by e.g., direct agonism/antagonism of these receptors, indirect effects via modulation of synthesis or clearance of endogenous receptor ligands, interference of the downstream signaling pathways or the endocrine feedback systems between tissues or through epigenetic changes that dysregulate the hormonal signaling pathways and may be transmitted to the next progeny [3]. Due to the high degree of sequence similarity and conservation of signaling pathways for many receptors across species, EDCs can exert their effects in both wildlife and humans. As the same receptors are often critical for the growth, maturation, and maintenance of endocrine tissues, the effects of EDCs are thought to be especially problematic for developing individuals. The adverse effects on birth outcomes, sexually determined physiological characteristics, reproductive health, and neuroendocrine functions in humans have been well documented in epidemiological studies [1,4].

Recently, EDCs have been linked to disturbances in metabolic processes such as type 2 diabetes (T2D), metabolic syndrome, obesity, and non-alcoholic fatty liver disease (NAFLD) that are increasingly prevalent in Western societies and among the younger populations. Several mechanisms proposed to underlie these aberrations include dysregulation of food and energy intake at the gut–brain axis, interference of normal energy consumption, abnormal storage of energy in adipose tissue (obesity) and the liver (steatosis), and unbalances in handling of energy sources between tissues [5,6]. Several NRs expressed in the liver coordinate the hepatic glucose, lipid bile acid and energy metabolism and their functions can be modulated by EDCs (Table 1.) [7,8]. Among these NRs, the peroxisome proliferator-activated receptors (PPARs), pregnane X receptor (PXR), and constitutive androstane receptor (CAR) seem to play central roles in glucose and fatty acid metabolism.

Here, we review the characteristics of CAR, mechanisms by which CAR is involved in metabolic processes, and finally, the effects of metabolism-disrupting chemicals on CAR activity and signaling. To this end, we selected the currently known EDC classes from existing literature since the year 2000 with an emphasis on compounds that have been associated with metabolic disruption. We then extended our literature searches on each EDC class and their prototypical compounds to identify articles showing interaction with CAR in a variety of in vitro, cellular, and animal models. Targeted searches based on reference lists, available Toxcast data, and other relevant NRs were also conducted. Thus, EDCs that interfere only with e.g., reproductive system and do not interact with CAR are not included here.

## 2. Key Characteristics of CAR and Its Activation Process

The constitutive androstane receptor (CAR, NR1I3) is a member of nuclear receptor subfamily 1I, together with its sisters vitamin D receptor (VDR, NR1I1) and pregnane X receptor (PXR, NR1I2), and a subject of intense research for the past 20 years. The many earlier review articles on CAR properties, target genes, evolution, and other aspects have been listed previously [72] and later progress in the field is also well-documented (e.g., [73,74,75,76,77,78]). Therefore, we provide below only an overview on key characteristics of CAR.

CAR was initially identified as a constitutively active modulator of retinoic acid receptor signaling [79]. Subsequent discoveries established CAR as a liver-predominant, ligand-activated regulator of xenobiotic-metabolizing enzymes such as cytochrome P450s (CYPs), conjugating enzymes and transporters, and identified the main molecular mechanisms underlying this induction pathway. First, CAR activates the *Cyp2b10* gene transcription after exposure of liver cells to phenobarbital (PB) and other PB-like inducing drugs by binding as a heterodimer with retinoid X receptor (RXR) to a distal enhancer sequence termed PBREM [80,81]. During this activation, CAR is translocated from cytoplasm into the nucleus [82] after dissociation of CAR-interacting cytoplasmic partners such as heat shock protein and CCRP [83]. The translocation in turn involves inducer-dependent dephosphorylation of the receptor, which is mediated by protein phosphatase 2A that counteracts the action of protein kinase ERK1/2 [84,85].

Consistently with these molecular studies, CAR null mice do not display induction of drug metabolism, proliferation of endoplasmic reticulum, or liver growth in response to PB or many other rodent tumor promoters [86,87]. Differences in CAR protein sequences cause significantly distinct ligand profiles between species [72,88]. In addition, humans but not rodents have splicing isoforms that are differentially activated by ligands [89]. The robust hypertrophy of rodent liver to PB exposure seems to be missing in humans, perhaps due to differences in CAR target genes in these species [90].

Development of various in vitro assays, coupled with mutagenesis and structural models, have clearly established that diverse chemicals act either as direct agonists or inverse agonists of CAR [91,92,93,94]. However, some compounds including PB are called indirect activators due to their inability to elicit binding to CAR, as measured by e.g., recruitment of NR coactivators, despite the fact that these compounds can induce CAR target genes [95]. It should be also noted that many CAR-activating chemicals can bind to other NRs, especially to PXR and PPARs [96,97], and several NRs and CAR may crosstalk, notably via sharing of DNA binding sites by CAR and PXR [72], PPARα [98], and hepatocyte nuclear factor-4 (HNF4; [99]).

CAR-mediated induction is, at least for PB, associated by the attenuation of epidermal growth factor (EGF) signaling [84,100]. Additionally, insulin and CAR pathways have been reported to intersect [101]. Finally, there is evidence that other NRs [72], genetic background [102], fasting [103], and aberration of the circadian clock [104] may enhance CAR expression levels. These interactions provide additional mechanisms to integrate glucose metabolism, energy utilization, liver proliferation, and xenobiotic metabolism.

## 3. Metabolic Effects Modulated by CAR

**Figure 1:**
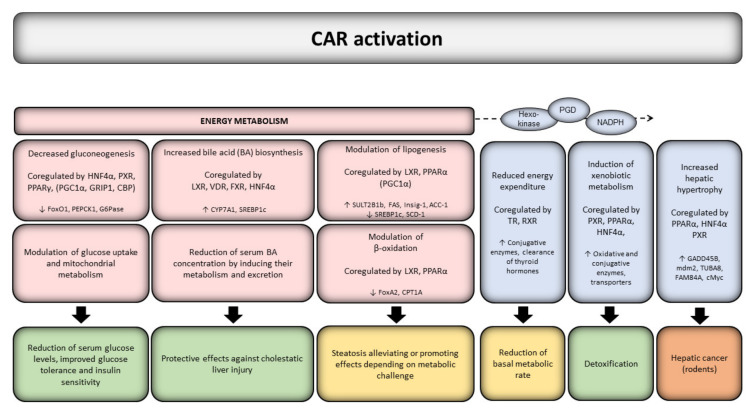
Schematic overview of key regulatory processes affected by CAR. CAR activation modulates key metabolic processes, such as glucose and lipid metabolism and bile acid synthesis via several mechanisms and pathways (as detailed in the main text). These effects depend in part on sex, nutritional status, or metabolic challenge used in animal studies. CAR-mediated induction of genes involved in glucose uptake and utilization (e.g., hexokinase, PGD) generates NADPH, which in turn supports xenobiotic metabolism. Through regulation of thyroid hormone levels, CAR can contribute to energy expenditure and weight loss. CAR alters the expression of genes associated with cell proliferation and oncogenic signaling in rodents. Positive outcomes are depicted with green, ambivalent outcomes with yellow, and adverse outcomes with orange color.

The many CAR-responsive genes code for proteins involved in disposition of drugs and xenobiotics, cell cycle control, and in endogenous metabolic processes [75,105]. Evidence from in vivo animal and in vitro human studies indicates that CAR is actively controlling key energy metabolism processes, such as hepatic glucose and lipid metabolism and is involved in the pathogenesis of metabolic disorders [72,73,106] (Figure 1). CAR has been reported as a key mediator in protecting against steatosis via suppressing lipogenesis and gluconeogenesis, and further, activation of CAR protects against fatty liver [106]. CAR has also been shown to participate in thyroid hormone metabolism [107,108,109].

The effects of CAR on metabolic homeostasis are mediated e.g., by reduced expression of several factors associated with energy expenditure, fatty acid synthesis, β-oxidation, bile acid synthesis, and gluconeogenesis [110,111] (Figure 1). CAR has been connected to metabolic stress during long-term fasting in mice by downregulation of triiodothyronine (T3) and tetraiodothyronine (T4) through the induction of *Sult1a1, Sult2a1,* and *Ugt1a1* genes, thus reducing the basal metabolic rate [112]. Additionally, CAR is required for the PB-induced decrease in T3 and T4 levels [113]. CAR further competes with the thyroid hormone receptor (TR) for binding to the mutual heterodimerization partner retinoid X receptor (RXR)α and NR coregulators [98] and may thus reduce the effects of thyroid hormones by decreasing the ability of TR to regulate its target gene expression.

Activation of CAR has been shown to repress gluconeogenesis [114,115] and to reduce serum glucose levels [115] in mice, and to lower lactate production and glucose consumption [116,117] in HepaRG cells. After a meal, insulin prevents hepatic glucose output by repressing phosphoenolpyruvate carboxykinase-1 (*PEPCK1*) and glucose 6-phosphatase (*G6Pase*) transcription. Upon fasting, *G6Pase* and *PEPCK1* genes are reactivated by the transcription factor forkhead box O1 (FoxO1) to initiate gluconeogenesis in the absence of insulin. Mechanistically, CAR can bind and repress FoxO1 [118], preventing it from interacting with the insulin response elements in insulin-like growth factor-binding protein (IGF-BP) 1, pyruvate carboxylase (PCX), fructose bisphosphatase 1 (FBP1), PEPCK1 and G6Pase upstream regions, and thus, suppressing the expression of these gluconeogenic genes [119]. CAR can compete with HNF4α for binding to the *PEPCK* promoter [99] or for metabolic coregulators, such as peroxisome proliferator-activated receptor γ coactivator-1 (PGC1)α or glucocorticoid receptor-interacting protein 1 (GRIP1) [110,120]. Further, CAR can suppress hepatic gluconeogenic gene expression through posttranslational regulation of degradation and subcellular localization of PGC1α, representing a possible cellular adaptive mechanism in energy-restricted conditions [121]. Activation of CAR by TCPOBOP has been shown to decrease glucose transporter (GLUT) 2 expression in wild-type but not in CAR null mice [110], indicating a reduction of hepatic glucose uptake, which may lead to inhibition of glycogenesis and stimulation of glycogenolysis.

CAR activation also enhances mitochondrial metabolism and increases bile acid production, lactate elimination, and glucose production [116,117,122], resulting in improved glucose tolerance and insulin sensitivity [115,121]. Agonist-activated CAR has been shown to improve insulin sensitivity in high fat diet (HFD)-fed and genetically obese *ob/ob* mice, while CAR null and antagonist-treated mice are resistant to insulin [57,123]. In early human studies, PB appeared to lower fasting plasma glucose and insulin levels, improve glucose tolerance and insulin response to glucose loading without affecting the body weight in diabetic patients [124,125]. Similar effects are seen in mice, where TCPOBOP-activated CAR prevents or ameliorates obesity and improves T2D symptoms induced by HFD in wild-type mice [115,126]. PB has later been shown to act as an insulin receptor antagonist and to elicit both CAR-independent increases and CAR-dependent decreases of blood glucose levels in wild-type and CAR null mice [101]. Long-term CAR activation in mice increases glucose uptake and utilization in the liver [115], by upregulating glucose transporters, glycolytic and mitochondrial pyruvate-metabolizing genes, and glycolytic intermediates in the liver [127]. Fasting and caloric restriction increases the activity of certain metabolic pathways, which may be regulated by CAR without exposure to exogenous agonists or activators [103,128]. The fasting-induced expression and activation of CAR has been shown to be controlled by the interplay of at least PPARα, HNF4α and PGC1α [129,130], implying a feedback regulation of glucose levels. Recently, the growth arrest and DNA damage-inducible gene 45b (*Gadd45b*), associated with liver growth (Figure 1), is required for anti-diabetic and obesity effects of CAR in vivo [131] but deciphering the exact molecular mechanisms requires further studies.

The effects of CAR to lipid metabolism are more controversial. Overall, several studies have shown that modulation of CAR may lead to changes hepatic triglyceride levels and thus, constitutes an important adverse outcome pathway (AOP) in metabolic effects of xenobiotic compounds [129,132]. In rodents, several studies have reported CAR as a key mediator in protecting against steatosis via suppressing lipogenesis and gluconeogenesis and further, activation of CAR protects against fatty liver [133]. Activation of CAR has been shown to alleviate hepatic steatosis and fatty liver by inhibiting hepatic lipogenesis and inducing β-oxidation in HFD-fed and TCPOBOP-treated mice [115,126]. Treatment of hyperlipidemic mice with CAR agonists decreases hepatic content of cholesterol by enhancing its metabolism to bile acids [134]. Activation of CAR reduces serum bile acid concentrations through induction of expression of genes, such as *CYP*s, *UGT*s, and *SULT*s, involved in bile acid metabolism and excretion [135,136]. In mice, CAR is involved in the regulation of enzymes producing bile acids [137] and its activation protects against cholestatic livery injury. Conversely, CAR activation contributes to increased lipogenesis, increased circulating fatty acid and ketone bodies, and represses β-oxidation [111,114,115,133]. Further, in primary hepatocytes, CAR activation did not affect the expression of lipogenic genes [138]. This discrepancy may be in part due to differences in the metabolic challenge and regulation of multiple pathways in diverse experimental settings. It should be noted that e.g., HFD feeding influences the expression/activation of NRs and their target genes [139]. Mechanistically, CAR has been shown to affect the insulin-induced gene-1 (INSIG1) and suppress lipogenic gene expression [115,129,140]. Further, activation of CAR in wild-type mice, but not in CAR/PXR null animals, leads to downregulation of PPARα and its target genes, such as carnitine palmitoyltransferase-1 (CPT1) that is involved in fatty acid oxidation [141]. This may be caused by CAR-dependent repression of FoxA2 and HNF4α transcription factors. CAR modulates the expression of fatty acid synthase (FASN), acetyl-CoA carboxylase 1 (ACC1), and stearoyl-CoA desaturase 1 (SCD1) that control de novo fatty acid biosynthesis [119]. While the expression of FASN and ACC1 increased after CAR activation in wild-type mice, the expression of SCD1 and the sterol regulatory element-binding protein-1c (SREBP-1c) was significantly decreased, suggesting complexities in regulatory networks. Interestingly, SREBP-1 seems to prevent interaction of coregulators with CAR, thereby inhibiting transcriptional activity of CAR and the expression of its target genes [142].

According to recent studies, at least some of the CAR-mediated metabolic effects are sex-dependent [143,144,145]. While CAR null male mice develop a range of metabolic disorders (obesity, insulin insensitivity, glucose intolerance, dyslipidemia, and liver steatosis), these symptoms were either mild or not observed in CAR null females [143]. However, female CAR null mice developed more severe symptoms after ovariectomy. Similar dimorphic effects have been observed in rats, with males manifesting with increased liver weight, reduced serum T4, and decreased serum total cholesterol, while females were unaffected [145]. Maternal CAR activation has been shown to improve glucose tolerance and to ameliorate gestational hyperglycemia and increase fetal weight in HFD-fed mice [146].

Even though the role of CAR in regulation of energy homeostasis and adverse metabolic effects has been established in rodents, many issues are still unclear. In contrast to classical hormone receptors, wide species differences in the ability of chemicals to modulate CAR activity cause significant challenges in predicting or understanding the metabolic consequences of CAR modulation by EDCs in humans [138,147,148]. As an example, CAR activation in mouse primary hepatocytes repressed the expression of genes involved in gluconeogenesis, lipogenesis, and fatty acid synthesis, activation of CAR in human primary hepatocytes inhibited gluconeogenesis without suppressing fatty acid synthesis.

Considerable crosstalk between CAR and other NRs and transcription factors that regulate lipid metabolism (PXR, FXR, PPARs, and LXR) [114] and glucose metabolism (PXR, HNF4, CREB, and FOX proteins) [149] lead to complex regulatory networks. Additionally, the metabolic enzymes and transporters activated by CAR are involved in clearance of metabolically relevant endogenous substances that include ligands of other NRs (bile acids, bilirubin, vitamin D, and thyroid hormones) (e.g., [113,114,150,151]). The lack of human CAR-selective tools adds to the challenge of deciphering the detailed role of CAR in metabolic processes. The 6-(4-chlorophenyl)imidazo[2,1-b][1,3]thiazole-5-carbaldehyde-O-(3,4-di-chlorobenzyl)oxime (CITCO) is routinely used as a positive control substance selective to human CAR. Recently, it was shown to bind and activate human PXR in hepatic cell models [152]. Further, as humans are continuously exposed to a large variety of EDCs, which may act in additive or synergistic ways, deciphering the net effects and connecting a specific EDC to specific regulator and metabolic alteration is a challenging task.

## 4. Metabolic Effects of EDC Classes Potentially Mediated by CAR

Below, we present evidence of main EDC classes as CAR activators and their potential metabolic effects (Table 2). It should be noted that a direct role of CAR in metabolic disruption, especially for humans, cannot be easily determined due to lack of highly selective CAR agonists, promiscuity of many EDCs for many NRs and other targets (Table 1), the complex interplay between NRs in controlling the hepatic metabolism, and species differences among animal species and with humans.

### 4.1. Bisphenols

These precursors to polycarbonate and vinyl ester plastics are of concern due to high affinity of some bisphenols to estrogen receptors [181] and their association with childhood and adult obesity [4,154] (Table 2). In vitro studies have suggested that exposure to bisphenol A (BPA) can cause metabolic dysfunction in adipocytes [182], while early exposure has been shown to cause weight increase particularly in female rats [183]. In epidemiological studies, BPA has been shown to affect insulin synthesis and release as well as insulin signaling [184]. In vitro NR binding assays indicated a very high affinity (<20 nM) of several bisphenols such as bisphenol A, AF, B, and C for human CAR [185], which was similar to their affinity for estrogen receptors (Table 2.). In contrast, human PXR bound these bisphenols only at micromolar concentrations. In agreement with this study, bisphenols A, B, and AF were among the most effective CAR activators in the yeast two-hybrid assay [186], and bisphenol A was a strong activator of human CAR1 and CAR3 isoforms while human CAR2 and PXR were refractory [11]. In addition, tetrabromobisphenol A is a flame retardant that appears to weakly activate CAR and reduce thyroid hormone levels in subacute toxicity studies rats [155], although similar dosages in other studies have not resulted in CYP2B induction [187] (Table 2). The higher propensity of CAR activation as compared to other hepatic NRs suggest a connection with the metabolic disorders associated with bisphenols.

### 4.2. Phthalates

They are a widely used group of plasticizers present in containers, coatings, tubes, and in myriad of other household appliances. Di(2-ethylhexyl)-, diisononyl-, and dibutyl-phthalates (DEHP, DINP, DBP) are among the most commonly used, and as esters, phthalates are easily hydrolyzed to their monoester derivatives MEHP, MINP, and MBP. Epidemiological studies have associated exposure to phthalates not only to maturation but also to increased risk of childhood obesity, diabetes, and impaired glucose tolerance [4,157] (Table 2). Mechanistically, the adverse effects on lipid metabolism and obesity could be caused by activation of PPAR isoforms in several tissues [188,189]. However, di- and monoester phthalates activate human and mouse CAR and PXR in reporter gene assays [11,23,24,25] (Table 2). Rodent models [156,158,190] also show evidence of CAR- and PXR-dependent activation. In addition, activation of CAR seems to downregulate and suppress PPAR-mediated signaling [141,191] (Table 2). These findings may contribute to phthalate-associated disturbances in glucose homeostasis.

### 4.3. Perfluoroalkylated Substances (PFAS)

These compounds, typified by perfluorooctanoic acid (PFOA) and perfluorooctane sulfonate (PFOS), have numerous uses in consumer and industrial products as e.g., surfactants and water-repelling coatings. They accumulate in organisms due to their very slow metabolism, raising therefore concerns about toxicity [192]. More recently, a strong association with childhood adiposity, adult obesity and impaired glucose tolerance in human cohort studies was reported [4]. The risk of thyroid disruption is estimated low [193] and linkage between sex hormone-dependent effects weak or non-consistent (e.g., [194,195]).

Early rodent studies found that PFOA and PFOS exposure leads to activation of PPARα-controlled target genes but also to a strong induction of CYP2B and CYP3A mRNAs in a CAR-dependent fashion [162,163], similarly to some phthalates (Table 2). Shorter-chain fluorinated carboxylic and sulfonic acids [57,145] also induced CAR target genes although negative findings have also been published [196]. The mode of CAR activation is not clear as some reports suggest either a direct [197] or an indirect mechanism [28], or no effect in reporter assays [198]. It is of interest that CAR and PPAR signaling pathways are mutually suppressive [105,199].

One of possible metabolic effects mediated by PFOA/PFOS-activated CAR is the decrease in serum glucose [163] (Table 2). The increases in fatty acid oxidation is more likely governed by PPARα while accumulation of triglycerides [160,200] could in part be due to inhibition of PPARα signaling by activated CAR [75]. However, PFOA seems to decrease the severity of preexisting fatty liver disease [161], a process that is modulated by CAR. More research in PFOA/PFOS-mediated metabolic effects and involvement of CAR, especially in human systems, are needed.

### 4.4. Flame Retardants

Flame retardants are widely used in textiles, furniture, electronics, and other plastic-based industrial or consumer products. The main classes are brominated diphenyl ethers (BDEs) and organophosphates, both of which are of concern for their endocrine-disruptive effects [4,201]. With respect to potential CAR-mediated effects, both BDE-47 and BDE-209 are mouse and human CAR activators, and they increase serum glucose levels [31,32,33] and T4 clearance [164] in animal models (Table 2). For organophosphates, human CAR is prone to activation of triphenyl, tricresyl, and isopropylated phenyl phosphates [202] (Table 2). Human PXR is also modestly activated while the responses of mouse CAR and PXR are weaker. A mixture of tris(1,3-dichloro-2-propyl)phosphate, triphenyl phosphate, and tricresyl phosphate activated CAR in the mouse model, concomitant with changes in leptin and insulin levels and energy intake [32]. The species differences in CAR activation potential of various BDE and organophosphate congeners [31,202,203], overlap with PXR activation, and scarce information on congener-specific metabolic effects complicate the interpretation of these studies.

### 4.5. Polychlorinated Biphenyls (PCBs)

This group of persistent organic pollutants has been associated with type 2 diabetes and obesity [204,205]. Earlier studies have showed that both human and rodent CAR and PXR are activated by non-planar PCBs [40,41,203] with a tendency of human CAR being activated efficiently and with a preference of CAR over PXR (Table 2). No clear structure–activity relationship with respect to CAR activation among PCB congeners has been identified [41,206]. Both planar (dioxin-like) and non-planar PCBs have been reported to attenuate EGF signaling in analogy to PB [100,168]. However, the lack of CYP2B induction by planar PCBs implies some divergence in signal transduction at the EGF receptor level. Finally, several PCBs seem to increase expression of CAR in a human hepatic cell line [207], suggesting that CAR expression is controlled by both exogenous and endogenous signaling [72] which raises the possibility of synergistic PCB action by both activation and induction of CAR.

With regard to metabolic effects, PCB153 is a CAR-activating compound that augments the hepatic steatosis and inflammation observed in HFD-fed mice [167] (Table 2.). Increases in blood glucose levels in male mice have also been noted [166] although PCB153-elicited induction seems stronger in females [57]. In a cross-generational study, F1 mice exposed to PCB153 in utero and during lactation showed decreased serum lipid levels and better glucose tolerance during a HFD challenge [208]. In addition, exposure to the PCB mixture (Aroclor 1260) has been associated with obesity and fatty liver disease (Table 2.). This PCB mixture seems to affect several metabolism-linked NRs by activating PXR and CAR isoforms, antagonizing PPARα [209], and influencing aspects of energy metabolism, including lipid oxidation, food intake, insulin sensitivity, and gluconeogenic gene expression in CAR- or PXR-dependent fashion in mice [169].

### 4.6. Pesticides

Several groups of pesticides have been linked to deleterious effects on neuroendocrine, reproductive, and immunological functions and dysregulation of energy metabolism [3]. The use of organochlorinated compounds such as dieldrin or endosulfan have largely been abandoned but both activate CAR and PXR in vitro, and increase expression of their CYP target genes in animals or in human HepaRG cells [45,46,210,211]. The now banned but environmentally persistent DDT, methoxychlor and their metabolites are also direct activators of both rodent and human CAR and PXR [186,210,212,213].

More recently, numerous pesticides have been screened for NR activation. Activation of human CAR and induction of CYP2B6 mRNA in HepaRG cells seems more pronounced than that of mouse CAR by many pesticides of the organophosphate, pyrethroid, and carbamate classes [44] (Table 2). Most pyrethroids tested activate mouse, human, and rat PXR in vitro, and activation was decreased by microsomal metabolism of these compounds [47,214]. A large number of organophosphates are also PXR activators albeit often in a species-dependent manner [214]. A prototypical triazine compound, atrazine, strongly induced CYP2B6 expression without any apparent human CAR activation [44] or EGF receptor binding [100], a finding that may be explained by increased expression of CAR [215] and contribution to CYP2B6 induction by atrazine-activated PXR.

Despite the strong evidence for CAR and/or PXR activation in cellular systems, these compound classes have mostly been investigated in animal studies for their hepatotoxic or tumor-promoting properties related to CAR (e.g., [87]) without a major focus on metabolic changes. Notably, recent reviews on endocrine disruptor-related metabolic processes [4,153,216] only mention the most persistent organochlorine compounds but not the more labile organophosphate pesticides.

There is limited concern on the reproductive adverse effects of azole fungicides in animal studies (e.g., [217,218]) while they often cause hepatocyte hypertrophy, liver toxicity, and even neoplasm formation in rodents (e.g., [219,220]). The activation of rodent and human AhR, CAR, and PXR by 20 different azole fungicides has recently been reviewed in detail [55] and we provide here only some examples (Table 2). Reporter gene assays and mRNA induction studies show that propiconazole is a moderate activator of human and rodent CAR. This is supported by mouse and rat studies in vivo [56,174]. At the same time, propiconazole activates PXR in all three species [55]. In contrast, tebuconazole appears to antagonize human CAR [56] while activating the rodent receptors. Coincidentally, tebuconazole-induced liver hypertrophy is not as highly dependent on CAR as with cyproconazole or fluconazole [172]. Vinclozolin is an anti-androgenic fungicide [221] that has been tested in Toxcast high-throughput analyses for NR activation; however, it does not show consistent activation of CAR or PXR [222]. A common metabolic outcome by azole fungicides in rodents is the frequent steatosis which is caused by activation of fatty acid synthesis, mostly via PXR and less frequently by CAR-mediated action [55,223]

### 4.7. Triclosan

Triclosan is an antimicrobial chlorinated phenoxyphenol used until recently in multiple personal care products, detergents, and technical equipment. Despite its weak affinity for steroid hormone receptors, there is little evidence for its adverse reproductive effects [224,225]. The response of CAR to triclosan seems highly species-specific: mouse CAR and human CAR3, a splice isoform with low basal activity, are activated by triclosan, while it is an inverse agonist for rat CAR and the main human isoform CAR1 [62,176,226] (Table 2). In a similar fashion, triclosan activated human PXR but not rat PXR [62], and mouse PPARα but not human PPARα [63]. These complex patterns may explain in part the inconsistent link between childhood obesity and triclosan exposure [225]. However, triclosan-induced decreases in T4 levels in the rat may be understood by CAR/PXR-mediated induction of T4-metabolzing enzymes [112,227].

### 4.8. Other CAR-Modulating EDC Classes with Limited Evidence for Metabolic Disruption

Alkylphenols are widely used as additives to lubricants and as precursors in chemical synthesis of e.g., polymers, surfactants, and detergents, such as alkylphenol ethoxylates which are degraded back to relatively persistent alkylphenols in the environment. Due to their mimicry of estrogenic substances, alkylphenols are thought to disrupt endocrine functions [228] (Table 2). Studies on their ability to modulate CAR are quite sparse. Nonylphenol activated mouse CAR in reporter gene assays and in mouse model albeit only in the females [13] which may be explained by the female-predominant expression of CAR and responsiveness of its target genes [229,230]. Surprisingly, nonylphenol did not activate CYP expression in PXR null mice where CAR is present, suggesting interplay between the two NRs [14]. Nonylphenol appeared to induce CYP2B genes in human primary hepatocytes and in humanized CAR mice [231] and later studies using the yeast two-hybrid assay demonstrated that many linear or branched chain alkylphenols, including nonylphenol, can activate human CAR at low to sub-micromolar concentrations [186]. Studies on alkylphenol-induced and CAR-mediated changes in metabolic processes have not been performed yet.

Parabens are *p*-hydroxybenzoic acid esters used as antibacterial preservatives in many types of consumer products. Although widespread, they are regarded as non-persistent due to their relatively rapid hydrolysis [232]. There is limited evidence for their endocrine effects although metabolic [180] (Table 2), and reproductive [233] outcomes have been reported. A recent study [60] tested 17 different parabens in NR activation assays at low micromolar concentrations. They found that rat CAR was weakly activated (2-fold) by butyl- and isobutylparabens, inverse agonism was seen with longer pentyl- and hexylparabens, and butylparaben-mediated activation was abrogated upon metabolism. Rat PXR, human PXR, and rat PPARα were also modestly responsive to various paraben derivatives. Unfortunately, mouse and human CAR were not tested. Human CAR was activated weakly by linear parabens in a yeast two-hybrid assay [186]. The high concentrations used, the narrow range of activating ligands, and their metabolic lability suggest that parabens are not likely potent CAR-dependent metabolic modulators, although definitive studies are lacking at the moment.

### 4.9. Other CAR-Modulating Compounds

A large repertoire of pharmaceuticals are CAR activators [72,94], while information on their effects on energy metabolism is largely unknown, and not further analyzed in this work. As one example, exposure to statins is a known risk factor for type 2 diabetes (e.g., [234]). Although statins can activate CAR modestly [235], they affect PXR more strongly [236]. Other mechanisms underlying the statin-elicited increase in blood glucose levels likely exist [237]. Similarly, traditional herbal medicines are often CAR/PXR activators [238] and there is some evidence for their efficacy in alleviation of some aspects in liver diseases [239,240]. Many flavonoids and natural estrogenic compounds are either direct or indirect CAR activators [177,241,242]. Some of them have been reported to improve glucose balance in diabetic mice [243] or reduce fatty acid accumulation in a CAR-dependent fashion [178], although downregulation of CAR target genes has also been noted [244]. Aryl hydrocarbon receptor (AhR) mediates the main effects of 2,3,7,8-tetrachlorodibenzo-*p*-dioxin (TCDD) and other dioxin-like compounds (e.g., [245]) which can impair glucose metabolism [246,247] and cause fat accumulation [248]. In addition, AhR activation may also lead to upregulation of CAR expression [249], perhaps as a secondary counteracting effect of the metabolic disruption. It is unclear if this is a direct transcriptional effect or due to AhR-mediated disruption of circadian regulation [250].

## 5. Future Directions

Metabolic processes in the liver are highly interconnected and subject to regulation by diverse signals that include e.g., neuronal control of food intake and satiety, circadian rhythms, production, use, and storage of glucose and lipids in the liver and inputs from other tissues such as intestine and muscle [5,6]. Nuclear receptors are only one part of this complex network. As evident from the previous sections, suspected metabolism-disrupting compounds are seldom specific for any one NR: for example, CAR activators are often ligands for PXR and PPARα, which also coregulate overlapping target genes [7]. There is a lack of sufficiently selective tool compounds that are both non-toxic and have favorable pharmacokinetics for teasing out CAR-dependent functions in normal human hepatocytes [8,152]. The interpretation of animals studies are fraught with complications due to large species differences between CAR and PXR ligand profiles and their target genes—this may not be easily alleviated even by the use of humanized mice [90] without careful and wide-ranging analysis of affected target genes. Further development of long-term human hepatocyte cultures, in connection of modern gene knockdown techniques, is likely to provide better and disease-relevant models for utilization in studies of CAR and PXR [251,252]. The effects of metabolic disruptors are affected not only by genetic and epigenetic variation among NRs and their target genes [253], but also by the type of diet that potentially predisposes or protects individuals from adverse effects [169,254]. Finally, studies on human-relevant mixtures of metabolic disruptors is largely missing [7].

## Figures and Tables

**Table 1:** Modulation of nuclear receptor activities by CAR-relevant EDCs.

EDC Group	Nuclear Receptors Relevant for Energy Metabolism [9,10]																	
	CAR		PXR		PPARs		LXRs		TR		ERRs		FXR	RXR	RAR	VDR	GR	MR
	h	r	h	r	h	r	h	r	h	r	h	r	h	h	h	h	h	h
Alkylphenols	↑ [11]	↑ [12]	↑ [13]	↑ [14]										↓ [15]				
Bisphenols	↑↓ [11,16]		↑ [16,17]		↑ [18,19]	↑ [18]		↑ [20]	↓ [21]	↓ [22]	↑ [17]						↓ [16,17]	↓ [17]
Phthalates	↑ [11,23,24,25]	↑ [11,23,24,25]	↑ [11,23,24,25]	↑ [11,23,24,25]	↑ [23]	↑ [23]	↑ [26]			↓ [27]								
PFAS	↑ [28]	↑ [28]			↑ [29]	↑ [30]												
Flame retardants	↑ [31,32,33]	↑ [31,32,33]	↑ [31]	↑ [31]	↑ [34,35]		↑ [26]		↓ [36,37]		↓ [38]			↑ [39]		↑ [39]	↑ [39]	
PCBs	↑ [40]	↑ [41]	↑ [40]	↑ [41]					↓ [42]								↓ [43]	
Pesticides	↑ [44,45]	↑ [44]	↑ [44,45,46]	↑ [44]	↑ [47]	↑ [48]			↓ [49]		↓ [50]	↓ [50]	↓ [51]		↑ [52]		↓ [43,53]	↓ [43,54]
Fungicides	↑↓ [55,56,57]	↑↓ [55,56,57]	↑ [55,56]	↑ [55,56]													↓ [58]	↓ [59]
Parabens		↑↓ [60]	↑ [60]	↓ [60]	↑ [61]	↑ [60]											↑ [61]	
																		
Triclosan	↑↓ [62]	↓ [62]	↑ [62]		↓ [63]	↑ [63,64]												
THMs, natural compounds	↑ [65,66,67]	↑ [67]	↑ [67,68]	↑ [67]	↑↓ [51,69]		↑↓ [70]				↑ [71]							

CAR, constitutive androstane receptor; PXR, pregnane X receptor; VDR, vitamin D receptor; PPAR, peroxisome proliferator-activated receptor; FXR, farnesoid X receptor; LXR, liver X receptor; ERR, estrogen receptor-related receptor; RAR, retinoic acid receptor; RXR, retinoid X receptor; TR, thyroid hormone receptor; GR, glucocorticoid receptor; MR, mineralocorticoid receptor; PFAS, per- and polyfluoroalkyl substances; PCB, polychlorinated biphenyls; THM, traditional herbal medicines; ↑ = activation/agonism, ↓ = inactivation/antagonism; h = human (in vitro), r = rodent (in vitro, in vivo).

**Table 2:** Examples of CAR related chemicals and their proposed metabolic effects via CAR.

Chemical Group	Example Compound (CAS)	Reported CAR Response	Toxcast AC50 (µM)	Metabolic Effect with Probable CAR Participation	References
Bisphenols	Bisphenol A, BPA (80-05-7)	Agonist	46.4 ^RG, (a)^ 20.3 ^RG, (b)^ 0.00892 ^BA, (c)^	Human: childhood and adulthood obesity ↑, diabetes ↑ Mouse: glucose tolerance ↓	[4,153,154]
	Tetrabromobis-phenol A, TBBPA (79-94-7)	Antagonist	29.3 ^RG, (b)^	Rat: thyroid hormone level ↓, reactive oxygen species ↑, bodyweight↑ at high dose levels	[155]
Phthalates	Dibutyl phthalate, DBP (84-74-2)	Antagonist	16.1 ^RG, (b)^ 16.2 ^BA, (c)^	Human: diabetes ↑, insulin resistance ↑ Rat fetus: steroid metabolism ↑	[4,24,156,157]
	Di-(2-ethylhexyl)-phthalate, DEHP (117-81-7)	Agonist (hCAR2)	inactive ^RG, (a), (b), BA, (c),^ ^RG, (d)^	Human: birth weight ↓ childhood and adult obesity ↑, diabetes↑, insulin resistance↑, glucose tolerance↓ Rat: fatty acid metabolism↑, tryptophan metabolism ↑	[4,156,158,159]
Perfluoro-alkylated substances	Perfluorooctanoic acid, PFOA (335-67-1)	Activator	18.7 ^BA, (c)^	Human: glucose tolerance ↓ adult obesity ↑, child adiposity ↑, diabetes↑ Mouse: hepatic steatosis ↓ Rat: mitochondrial respiration↓ Rat hepatocytes: mitochondrial respiration↓, energy metabolism ↓, fatty acid oxidation↑, hepatic triglycerides ↑	[4,28,106,159,160,161,162]
	Perfluorooctane sulfonate, PFOS (1763-23-1)	Antagonist	17.6 ^BA, (c)^	Human: adult obesity ↑ Rat: mitochondrial respiration ↓ Rat hepatocytes: mitochondrial respiration ↓, energy metabolism ↓, fatty acid oxidation ↑, hepatic triglycerides	[4,106,159,160,162,163]
Brominated ND organo-phosphate flame retardants	Polybrominated diphenyl ether 47, BDE-47 (5436-43-1)	Activator (mCAR, hCAR)	39.1 ^RG, (a)^	Human: obesity ↑ Mouse: thyroid hormone ↓, fasting glucose↑ (males), glucose clearance ↑ (females)	[31,32,154,164,165]
	Triphenyl phosphate, TPP (115-86-6)	Agonist	18.2 ^RG, (a)^	Mouse: bodyweight↓, energy intake ↓, ghrelin↑, leptin ↓, insulin ↓, fasting glucose ↑ (males)	[32]
	Tris(1,3-dichloro-2-propyl) phosphate, TDCPP (13674-87-8)	Antagonist	34.7 ^RG, (d)^ 0.586 ^RG, (b)^ 1.78 ^RG, (e)^	Mouse: bodyweight↓, energy intake ↓, ghrelin↑, leptin ↓, insulin ↓, fasting glucose ↑ (males)	[32]
PCBs	PCB153 (35065-27-1)	Activator	inactive ^RG, (a), (d)^	HepG2: EGFR signaling ↓ Mouse: blood glucose↑ (males), glucagon ↑ (females), diet-induced obesity ↑, non-alcoholic steatohepatitis ↑ (males), visceral adiposity ↑ *, hepatic steatosis ↑ *, β-oxidation ↓ *, lipid biosynthesis ↑ *, glucose tolerance	[40,100,166,167]
	Arochlor 1260 (11096-82-5)	Activator	inactive ^RG, (a), (d)^	Mouse: EGFR signaling↓, energy metabolism ↓, metabolic syndrome↑, insulin sensitivity ↑ *	[168,169]
Pesticides, insecticides	Dichlorodiphenyltrichloroethane, o,p’-DDT (789-02-6)	Agonist	4.05 ^RG, (a)^	Human: childhood obesity ↑ (prenatal exposure), adult diabetes ↑ (prenatal exposure), Mouse: glucose tolerance ↓ (perinatal and adult exposure), insulin secretion ↓	[4,153]
Fungicides	Cyproconazole (94361-06-5)	Agonist	30.2 ^BA, (c)^	Mouse: lipid accumulation ↑, altered fatty acid and phospholipid metabolism,	[170,171,172,173]
	Propiconazole (60207-90-1)	Agonist	48.2 ^RG,a)^ 16.4 ^BA, (c)^ 65.8 ^RG, (b)^	Mouse: liver weight ↑, fatty acid synthesis ↑, hepatic triglyceride accumulation↑, steatosis ↑, phospholipid degradation ↑, tryptophan metabolism ↑ Rat: fatty acid synthesis ↑, hepatic triglyceride accumulation↑, steatosis ↑	[55,57,174,175]
Triclosan	Triclosan (3380-34-5)	Agonist (hCAR3) Inverse agonist (hCAR, mCAR, rCAR)	4.71 ^RG, (b)^ 85 RG, (d)	Human: birth weight ↓ Mice: body weight ↓ (during exposure) Rat: hepatic catabolism of thyroid hormones ↑, thyroid hormone ↓	[4,62,176]
Natural compounds, phyto/myco- estrogens	5,7-OH flavone, chrysin (480-40-0)	Agonist (mCAR) Activator (hCAR)	39.1 RG, (a)	Mice: detoxification ↑, energy metabolism↑, fatty acid accumulation↓ in mouse livers with alcohol-induced stress. A431 cells: inhibit EGFR autophosphorylation at Tyr1068	[177,178]
Alkylphenols and derivatives	Nonylphenol (104-40-5)	Agonist	68.7 ^RG, (a)^	*Daphnia magna*: testosterone elimination ↓, reduced/dehydrogenated testosterone metabolites ↑, androgen accumulation ↑	[13,14,179]
Parabens	Butylparaben (94-26-8)	Agonist	36.5 ^RG, (a)^ 60.2 ^RG, (b)^	Human: adverse cardiometabolic effects, blood glucose ↑ (pregnant women), diabetes ↑, obesity ↑	[180]

hCAR = human CAR, mCAR = mouse CAR, rCAR = rat CAR. AC50 = chemical concentration where 50% of the maximum response is achieved. RG = reporter gene assay, BA = binding assay, (a) TOX21_CAR_Agonist = increase in CAR-dependent luciferase reporter activity, (b) ATG_PBREM_CIS_up = RT-PCR-based measurement of PBREM-driven reporter gene mRNA induction, (c) NVS_NR_hCAR_Antagonist = loss of FRET signal from cell-free coactivator/CAR interaction, (d) TOX21_CAR_Antagonist = decrease in CITCO-activated CAR-dependent luciferase reporter activity, (e) ATG_CAR_TRANS_dn = RT-PCR-based measurement of GAL4-CAR-mediated reporter gene mRNA induction.
